# Juvenile Dermatomyositis (JDM) Complicated by Thrombotic Thrombocytopenic Purpura (TTP) and Purtscher's Retinopathy Responsive to Rituximab: Case Report and Literature Review

**DOI:** 10.3389/fped.2020.00436

**Published:** 2020-08-04

**Authors:** Keerthi Gullapalli, Ofra Goldzweig, Kabita Nanda, Ravi Chekka, Shanail Berry, Hulya Bukulmez

**Affiliations:** ^1^Internal Medicine, Sparrow Hospital, Michigan State University, Lansing, MI, United States; ^2^Pediatrics, Kaplan Medical Center, Rehovot, Israel; ^3^Rheumatology, Seattle Children's Hospital, Seattle, WA, United States; ^4^Pediatrics, Pediatric Care Center, Erie, PA, United States; ^5^Pediatrics, Metro Health System, Cleveland, OH, United States; ^6^Division of Pediatric Rheumatology, Department of Pediatrics, MetroHealth Medical Center, CWRU, Cleveland, OH, United States

**Keywords:** juvenile dermatomyositis, TTP, purtscher's retinopathy, ADAMTS13, rituximab, vWF

## Abstract

Juvenile dermatomyositis (JDM) is a multisystem vasculopathy that infrequently presents with acute complications (1). We report here the case of a 12-year-old girl with JDM who developed Thrombotic Thrombocytopenic purpura (TTP) and Purtscher's retinopathy. This is the second pediatric case of JDM with TTP and Purtscher's retinopathy in the literature. The diagnosis of JDM was based on her clinical presentation (fever, myalgia, proximal muscle weakness, characteristic skin rash and elevated muscle enzymes) (2). Despite improvement of rash, fever and weakness with corticosteroids and intravenous Immunoglobulins (IVIG), the patient developed retinopathy, thrombocytopenia, hemolytic anemia, renal failure, and pulmonary edema within 1 week of initial treatment. A clinical diagnosis of TTP and Purtscher's retinopathy was made and her ADAMTS13 activity was found to be low. Regardless of aggressive treatment with pulse steroid therapy, IVIG, plasmapheresis along with multiple infusions of Fresh Frozen plasma (FFP), her condition deteriorated. In view of her worsening condition, she received one dose of Rituximab and within 48 h, her hematological and retinal involvements improved. Rituximab was given at the same dose once weekly thereafter for 4 total doses. Her disease process was halted, and retinopathy improved significantly in 48 h and continued to gradually improve over 3 weeks of maintenance therapy with cyclosporine, methotrexate, and IVIG and then stabilized. This report documents the association of TTP and Purtscher's retinopathy with JDM, emphasizing that early recognition and prompt treatment with rituximab along with the current standard of care treatment i.e., Vincristine, corticosteroids and plasmapheresis could be of potential benefit in controlling disease activity.

## Background

JDM is a rare autoimmune multi-system vasculopathy occurring in about 2–4 per Million children per year in the United States with peak onset between 5 and 14 years of age (1). Dermatological and muscle manifestations are most common at presentation. The diagnostic criteria for JDM includes: symmetric weakness of proximal muscles, characteristic dermatological changes (heliotrope discoloration of the eyelids with periorbital edema, and Gottron's papules which are erythematous scaly rash over dorsal aspects of the metacarpophalangeal and proximal interphalangeal joints), elevation in one or more serum skeletal muscle enzymes [creatinine kinase (CK), Aldolase, Aspartate Aminotransferase (AST), Lactate Dehydrogenase (LDH)], electromyographic demonstration of myopathy, muscle necrosis, perifascicular atrophy and inflammation on muscle biopsy (2). The pathogenesis of the disease includes an autoimmune angiopathy with cell mediated immunity to muscle antigens. The cellular infiltrate includes a large component of plasmacytoid dendritic cells. Several autoantibodies are associated with JDM including both myositis-specific and myositis associated (2). Furthermore, von Willebrand factor (vWF), an endothelial bound clotting factor, is found to be elevated during JDM activity due to the inflammation and ongoing autoimmune vascular injury (3). It is unknown whether vWF has a role in triggering TTP. Although rare, adult onset dermatomyositis and TTP has been reported previously to be seen concurrently (4–10). There has been one report of a JDM patient from France that developed TTP and Purtscher's retinopathy (11).

TTP is a thrombotic microangiopathy with an annual incidence of 3–11 cases per million, with about 5–10% of the cases occurring in children (12). The disease is characterized by formation of microthrombi in multiple organ systems causing sequelae of hemolytic anemia, thrombocytopenia, renal injury, neurological changes and multiorgan dysfunction (12). The basic pathogenesis results from an imbalance between Ultra Large von Willebrand Factor (ULvWF) multimers and ADAMTS13 (a disintegrin; a metalloprotease with 13 thrombospondin type 1 repeats) either secondary to decreased production or the formation of antibodies against ADAMTS13 (12). ADAMTS-13 is a member of proteases with specific features involved in cleaving vWF multimers. ULvWF multimers are suggested to be cleaved by ADAMTS-13 at position 842Tyr-843Met preventing them to become multimers (12). The vascular thrombi are caused by intravascular accumulation of large multimers of vWF. Abnormalities of vWF protease activity are not restricted to patients with the diagnosis of TTP (13). TTP is a known complication of several autoimmune and inflammatory diseases including systemic lupus erythematosus (SLE) and dermatomyositis (DM). It is speculated that autoimmune activity with excess B-cell response and IgG antibodies to ADAMTS13 are key triggers in the development of secondary TTP in SLE (14). Thus, there has been a debate whether immune suppressive therapies that suppress B cell activity might be beneficial in autoimmune disease associated TTP treatment (15). Rituximab is an anti-CD20 antibody and has been found to be effective in autoantibody mediated autoimmune diseases including, autoimmune hemolytic anemia, thrombocytopenia, cold agglutinin disease (16) and acquired factor VIII inhibitors (17). Rituximab depletes the CD-20 positive B cells using antibody dependent cellular cytotoxicity and complement mediated lysis. Depletion of peripheral B cells might interrupt the ongoing humoral autoimmune response providing a rationale for its use in TTP (18). Although Rituximab treatment for TTP has been reported previously (5), children with JDM associated TTP who responded to rituximab have not been reported before.

Purtscher's retinopathy is a hemorrhagic and vaso-occlusive vasculopathy which was originally described as a syndrome of sudden blindness after head trauma but later described with pancreatitis and collagen vascular diseases (19). It is characterized by appearance of retinal whitening and hemorrhage and retinal edema predominantly around the optic disc (19). It is speculated to be caused by complement activation after acute crushing injuries to head or thorax (19). Here we describe a case, which presented with JDM and developed TTP, end organ damage and Purtscher's retinopathy that has shown a dramatic response to B cell ablating therapy with rituximab.

## Case Presentation

A previously healthy, athletic 12-year-old girl presented with a 2-week history of headache, febrile myalgia, muscle weakness, and rash (not shown). On examination, she had tenderness and weakness of proximal muscles of legs and arms, heliotrope rash, Gottron's papules, erythematous rash on trunk and upper arms (Shawl sign), and mild swelling of bilateral knees and ankles. Laboratory work-up demonstrated elevated muscle enzymes as shown in Table 1. A subsequent MRI of bilateral quadriceps demonstrated increased scattered heterogeneous T2 signal intensity indicating inflammation (not shown). The patient was diagnosed with JDM per Bohan and Peter criteria (2). On Eye examination, patient had 20/20 vision and fundus was normal.

**Table 1:** Key laboratory values upon 1st admission and readmission to PICU.

**Test**	**Normal**	**1st admission**	**PICU day1**	**PICU day2**	**PICU day3**	**PICU day6**
Aldolase (U/L)	1–7.5	37	67	–	–	–
LDH (U/L)	110–295	802	2,543	–	–	–
CK (U/L)	30–170	69	61	–	–	–
Creatinine (mg/dl)	0.7–1.3	1	2.9	3.3	4.4	5.3
BUN (mg/dl)	8–20	23	66	76	94	83
vWF (%)	55–200	418	382	–	–	–
AST (U/L)	8–40	15	92	–	–	–
ALT (U/L)	8–40	29	68	–	–	–
ADAMTS13 (%)	50–160	–	64	–	–	–
Factor VIII (%)	50–150	293.9	–	–	–	–
Ristocetin cofactor (%)	50–200	314	–	–	–	–
Hemoglobin (g/dl)	12-16	11.6	8.4	6.4	7	10.9
Platelets (per mm^3^)	150,000–400,000	301,000	61,000	34,000	–	15,000
PT (s)	11–15	–	13.5	–	–	–
PTT (s)	25–40	–	20.6	–	–	–
INR	<1.1	–	1.2	–	–	–
Fibrinogen (ug/dl)	150–400	–	350	–	–	–

Patient was hospitalized for pulse IV methylprednisolone (30 mg/kg/day) and one dose of IVIG (1 g/kg/day) treatment. Her laboratory values upon first admission are shown in Table 1. C-ANCA P-ANCA, Coombs test, anti-platelet antibodies, and ANA were negative. Weakness and rash improved significantly after treatment and she was discharged with oral prednisone (2 mg/kg/day) with taper in 4 weeks, methotrexate (20 mg/m2/week), folic acid (1 mg daily), and naproxen (20 mg/kg/day).

Despite rapid fever resolution and improvement of myalgia and muscle weakness, the patient presented to Emergency department approximately 1 week later with blurry vision and severe headache. The patient was admitted to Pediatric Intensive Care Unit (PICU). Course of her hospital stay, and medications given are as follows:

Day 1: Labs demonstrated exacerbation of JDM with elevated muscle enzymes, elevated acute phase reactants, anemia, thrombocytopenia, and renal failure as shown in Table 1. Peripheral blood smear showed schistocytosis. Treatment initiated with a pulse of IV methylprednisolone (30 mg/kg/d) and subsequently plasmapheresis was initiated. Patient also developed pulmonary edema which was managed with inhalation of bronchodilators, non-invasive ventilation and aggressive diuresis.

Day 2: Anemia, renal injury, and thrombocytopenia worsened (Table 1). Plasmapheresis was repeated, followed by FFP and another pulse of IV methylprednisolone (30 mg/kg/d).

**Figure 1:**
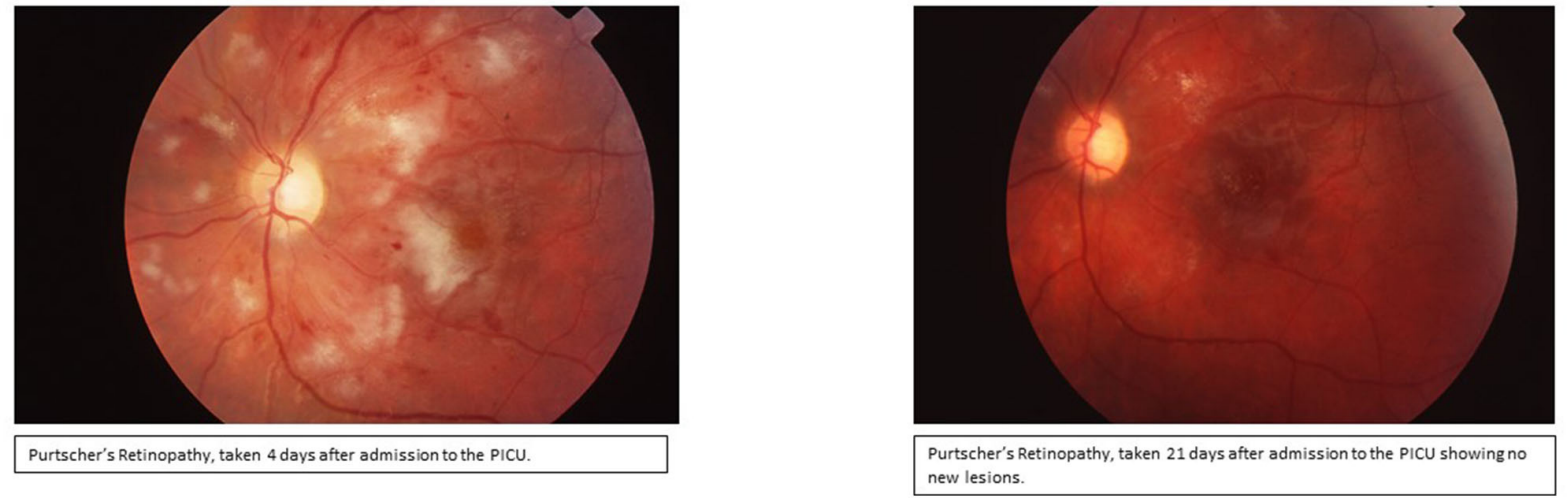
Ophthalmologic examination of fundus demonstrating confluent cotton wool spots around optic disc indicating Purtscher's retinopathy.

Day 3: Seizures developed, and she received Lorazepam 2 mg initially and then placed on Fosphenytoin (20 mg/kg/d). With the presence of renal injury, seizures, thrombocytopenia, hemolytic anemia, and schistocytes on peripheral smear, she was diagnosed with TTP. Hemolytic anemia and renal failure continued to worsen (Table 1). Another IV pulse methylprednisolone (30 mg/kg/d) dose was given and 2 volumes plasmapheresis were exchanged. Hemodialysis was performed and was given one-unit packed RBCs. Ophthalmologic examination demonstrated retinal perfusion abnormalities on angiography, whitening, Putscher flecken or cotton wool spots as shown in Figure 1. Patient diagnosed with Purtscher's retinopathy. No treatment was suggested by ophthalmology except for targeted therapy to underlying autoimmune disease. Patient was found to be homozygous for MTHFR 1298 and PAI-1 on a thrombophilia DNA assay panel and had no mutations on Factor V Leiden, Prothrombin 20210A, MTHFR 677, or Factor XIII V34L alleles.

Days 4: Renal injury, hemolytic anemia, and thrombocytopenia persisted. She received 2 cycles of plasmapheresis with FFP replacement, 2 cycles of hemodialysis, and two more methylprednisolone pulses.

Day 5: Vincristine 1 mg and IVIG 1 g/kg/d were given, no improvement was observed.

Day 6: Patients condition continued to worsen. B cell ablating treatment with rituximab 375 mg/m2/dose was initiated. She also continued to have hemodialysis (3 times a week), plasmapheresis (daily) with FFP, IVIG and IV methylprednisolone pulses.

**Figure 2:**
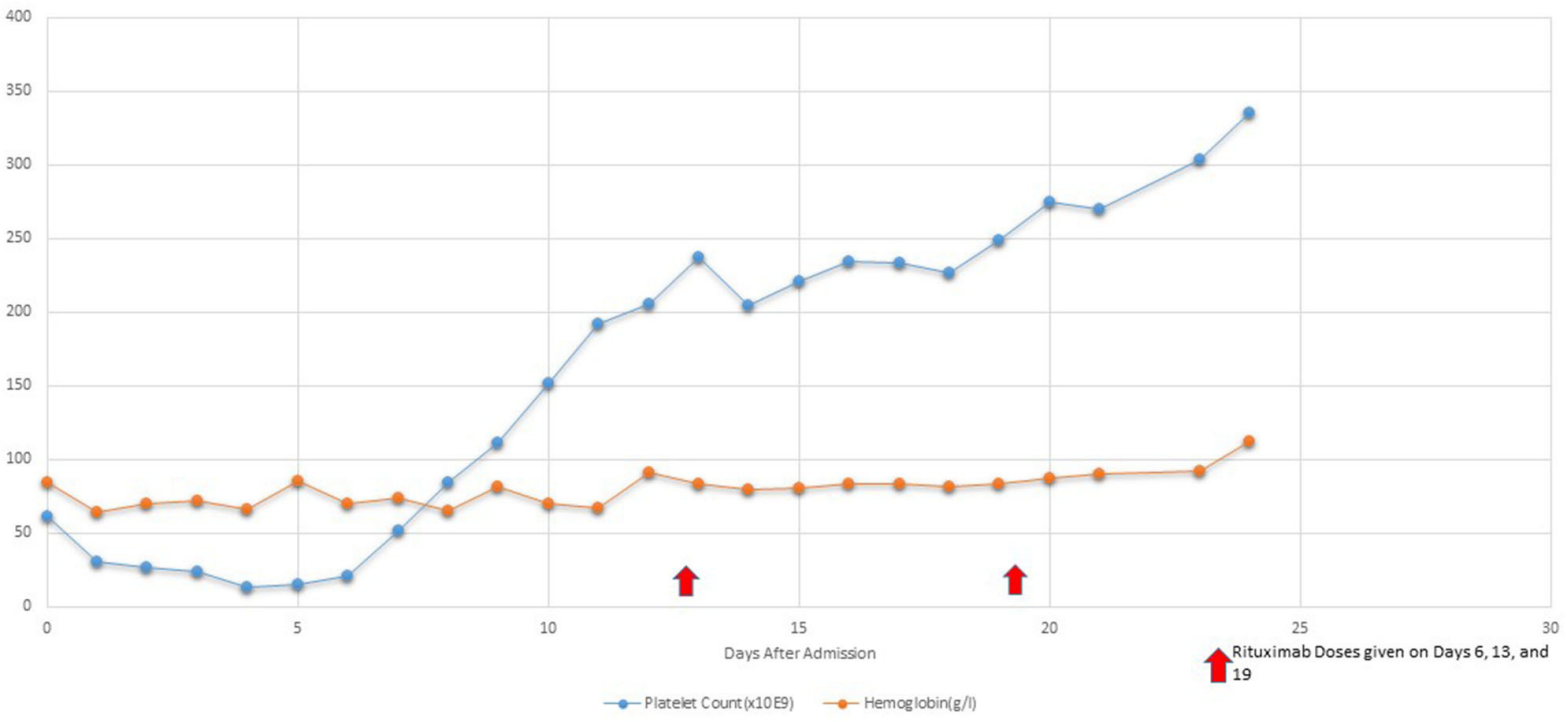
Platelet Trend in relation to Rituximab treatment.

After Rituximab treatment within 48 h patient's platelets (103,000/L) increased although renal injury persisted until hospital day 9. Her retinopathy improved significantly within 48 h of Rituximab treatment. Hemodialysis was discontinued on hospital day 9 due to significant improvement in renal function [BUN (39 mg/dL) and Creatinine (2.6 mg/dL)]. Patient continued to have 1 volume plasmapheresis/day until hospital day 11 with only two subsequent single volume plasmaphereses on hospital day 14 and 17. Subsequently, she received 4 total infusions of Rituximab (350 mg/m2/d) once weekly during her PICU stay (Figure 2). Her disease process was halted, and her condition continued to improve progressively. Figure 2 demonstrates the platelet trend in relation to Rituximab treatment.

On day 25, the patient was discharged home on Gabapentin and Methadone for pain, citalopram, Amlodipine, Atenolol, Levetiracetam, Phenytoin, and Granisetron, and was scheduled for regular outpatient IVIG infusions to maintain remission. For the following 2 years her retinopathy was followed up closely by a retina specialist and was observed to be normal while every 3 weeks IVIG treatments were continued.

Patient has been followed up by pediatric rheumatology till she turned 18 and her current condition is stable. She is attending school full time and continues to competitively swim. She can read using a magnifier and except for her ocular nerve atrophy the retinal lesions are all reported to be improved. She is being followed regularly by ophthalmology in her annual appointments.

## Literature Review and Discussion

In patients with DM occurrence of TTP is rare. We identified 9 cases of adult onset DM that subsequently developed TTP between 1985 and December 2018 (Table 2) (4–10). In all 9 previously published cases the mean age at TTP onset was 56 years, more prevalent in females. The interval from onset of DM to development of TTP was variable ranging from 1 week to several months. In terms of treatment, 7 out of 9 reported cases were given glucocorticoids and plasmapheresis was done in 7. Only 3 of 9 cases (33%) improved and survived. Only 1 case received Rituximab on day 19 but died due to septic shock on day 158 (5). The major cause of death in most cases was septic shock and multiorgan failure. Our patient received Rituximab early in course of disease (day 6) and started improvement within 48 h. We found only one adult DM patient who developed Purtscher's retinopathy (20). In JDM, there has been 1 case reported in France that developed TTP and Purtscher's retinopathy (11). Early IV corticosteroids with plasmapheresis improved the retinal findings back to normal in 6 months in that patient (11).

**Table 2:** Treatment and outcome of previously reported patients based on literature review.

	**Author**	**Year**	**Age**	**Treatment**	**Outcome**	**References**
1.	Malik et al.	2018	27/F	Plasmapheresis only	Improved	(4)
2.	Yamada et al.	2008	69/F	Methylprednisolone (mPSL), Tac, cyclophosphomide, IVIG, plasmapheresis	Improved on day 115	(5)
3.	Yamada et al.	2014	70/M	mPSL, IVIG, rituximab (day 19), plasmapheresis	Died on day 158 due to septic shock	(5)
4.	Yamada et al.	1996	65/F	Cyclosporine, IVIG, PSL, plasmapheresis	Died on day 74 due to septic shock	(5)
5.	Saito et al.	1993	42/M	IVIG, vincristine, corticosteroids, dextran, plasmapheresis	Improved	(6)
6.	Miyaoka et al.	1997	60/M	Plasmapheresis, mPSL, IVIG, antiplatelet therapy	Died on day 17 due to MOF.	(7)
	Shoda et al.	2009	57/M	mPSL	Died on day 13 due to respiratory failure	(8)
8.	Sawdyk and Jundt	1985	65/F	mPSL, plasmapheresis, antiplatelet therapy	Died on day 11 due to pneumonia	(9)
9.	Knox-Macaulay et al.	2004	48/F	mPSL, IVIG	Died immediately	(10)
10.	Bader-Meunier et al.	2012	7/M	PSL and plasmapheresis	Improved	(11)

Our patient's history of JDM with subsequent onset of TTP makes her one of the youngest patients reported to have developed both illnesses. Although both TTP and adult onset DM have been reported, a firm understanding of the relationship between the two disorders is not well-understood.

The most plausible explanation of DM followed by TTP is that excessive autoimmune activity due to B cells results in elevated amounts of vWF in the intravascular compartment which makes ultra large multimers and deposits on the vasculature. In addition, IgG autoantibodies are also produced toward ADAMTS-13 and decrease the cleavage of the vWF (14). Thus, during active disease, autoimmunity causes an imbalance between the vWF and ADAMTS-13 activity and triggers TTP onset.

In the present case with JDM while the immunoglobulin levels were high during the initial phase of the disease (not shown), ADAMTS-13 activity level was found to be low. Thus, we speculate that in our patient, ADAMTS-13 activity was not sufficient to cleave the elevated vWF during her active disease. Till date, a single optimal treatment approach has not been identified. Some of the most effective treatments include plasmapheresis alone or in combination with corticosteroids, vincristine, high doses of immunoglobulin. Splenectomy is the treatment option of last resort. IVIG is accepted as an effective treatment in JDM, particularly in those patients with severe vasculopathy (21). In our patient, the deterioration of her condition despite steroids, IVIG, vincristine, methotrexate, plasmapheresis, hemodialysis lead us to use Rituximab along with IVIG which improved her condition within 48 h. It is important to note that this B cell ablation therapy, which depleted immunoglobulin production and decreased the autoantibody production, halted the TTP process and has been successful in controlling the end organ damage in our case with JDM and TTP. Thus, we speculate that ongoing production of autoantibodies due to activated B cell function were primarily responsible for the TTP etiology.

Another pleasing outcome in our case was the improvement of Purtscher's retinopathy, despite what was predicted, during the PICU stay. In our patient, we speculate that retinopathy is a result of retinal vascular involvement resulting from TTP. IVIG has been suggested to be helpful in JDM vasculopathy and skin manifestations of JDM (21). IVIG might have been useful treating the retinal vascular damage in our case proving its value in treatment of microangiopathic end organ damage secondary to TTP. Thus, it is important to note that in retinopathy secondary to JDM with or without associated TTP, addition of IVIG to other chemotherapy may enhance the regeneration process. Future studies are needed to provide further proof of concept.

Physicians should keep in mind that TTP and/or Purtscher's retinopathy occasionally arise as serious complication of JDM as well as other autoimmune diseases. Furthermore, when VWF levels are found elevated, studying ADAMTS-13 activity might predict the microangiopathic thrombus development in JDM. Thus, anyone presenting with typical manifestations of JDM and clinical features suggesting TTP should be further investigated and treatment with combination of rituximab and IVIG in early stages of TTP needs to be considered in cases refractory to the standard of care.

In conclusion, we presented a very rare case of a patient with JDM with TTP and Purtscher's retinopathy. This case is important to discuss since it showed us the additive effect of Rituximab treatment in halting TTP process in JDM, and the successful improvement in Purtscher's retinopathy. Additionally, our review also indicated that the improvement of retinopathy in the presented case has been significantly shorter than any other reported.

## Data Availability Statement

All relevant data is contained within the article. Furthermore, all data supporting the conclusions of this article are available by the authors, without undue reservation, to any qualified researcher.

## Ethics Statement

Written and informed consent was obtained from the parents of the patient for the publication of this case report. Patient also gave us assent to report her case.

## Author Contributions

KG has been reviewing the case, literature and drafting the work for publication. OG partially drafted the work and reviewed the literature. KN agreed to be accountable for all aspects of the work in ensuring that questions related to the accuracy or integrity of any part of the work are appropriately investigated and resolved. RC has helped drafting the work, obtaining the ophthalmic pictures via collaboration, and he provided approval for publication of the content. SB has helped drafting the work, obtained history from the patient and collaborators, and revised it critically for important intellectual content, and provide approval for publication of the content. HB contributed substantial to the conception and design of the work, or the acquisition, and analysis or interpretation of data for the work. She also agreed to be accountable for all aspects of the work in ensuring that questions related to the accuracy or integrity of any part of the work are appropriately investigated and resolved. All authors contributed to the article and approved the submitted version.

## Conflict of Interest

The authors declare that the research was conducted in the absence of any commercial or financial relationships that could be construed as a potential conflict of interest.

## References

[1] Kwa MC Silverberg JI Ardalan K. Inpatient burden of juvenile dermatomyositis among children in the United states. Pediatr Rheumatol Online J. (2018) 16:70. 10.1186/s12969-018-0286-1 30424778 PMC6234588

[2] Cassidy J Petty R Laxer R Lindsley C Juvenile Dermatomyositis. Textbook of Pediatric Rheumatology. Elsevier (2005). p. 407–34. 10.1016/B978-1-4160-0246-8.50024-3

[3] Schwameis M Schorgenhofer C Assinger A Steiner MM Jilma B. VWF excess ADAMTS13 deficiency: a unifying pathomechanism linking inflammation to thrombosis in DIC, malaria. Thromb Haemost. (2014) 113:708–18. 10.1160/TH14-09-0731 25503977 PMC4745134

[4] Malik ZR Shahbaz A Aziz K Razaq Z Umair M Sachmechi I. Thrombotic thrombocytopenic purpura associated with dermatomyositis. Cureus. (2018) 10:e3161. 10.7759/cureus.3161 30357036 PMC6197508

[5] Yamada S Yamashita H Nakano M Hatano H Sasaki T Takahashi Y. Thrombotic microangiopathy with polymyositis/dermatomyositis: three case reports and a literature review. Intern Med. (2018) 57:2259–65. 10.2169/internalmedicine.0512-17 30068898 PMC6120848

[6] Saito Y Hamamura K Kurata Y Sugimoto T. Case of dermatomyositis complicated by thrombotic thrombocytopenic purpura (TTP) which responded to combination of gamma globulin and vincristine–clinical analysis on TTP cases in the Japanese literatures. Jap Jour of Clin Hemat. (1993) 34:68–73. 8450611

[7] Miyaoka Y Urano Y Nameda Y Shigekiyo T Horie T Sano N. A case of dermatomyositis complicated by thrombotic thrombocytopenic purpura. Dermatology. (1997) 194:68–71. 10.1159/000246062 9031797

[8] Shoda T Kotani T Takeuchi T Makino S Hanafusa T. A fulminant case of systemic sclerosis/dermatomyositis complicating thrombotic microangiopathy and diffuse alveolar hemorrhage. Nihon Kokyuki Gakkai Zasshi. (2009) 47:227–31. 19348271

[9] Sawdyk MA Jundt J. Dermatomyositis complicated by thrombotic thrombocytopenic purpura. Henry Ford Hosp Med J. (1985) 33:214–8. 4086331

[10] Knox-Macaulay HHM Adil SN Ahmed EME. Acute thrombotic thrombocytopenic purpura following doxycycline treatment of chlamydia pneumonia infection in a patient with dermatomyositis. Clin Lab Haem. (2004) 26:147–51. 10.1111/j.1365-2257.2004.00594.x 15053810

[11] Bader-Meunier B Monnet D Barnerias C Halphen I Lambot-Juhan K Chalumeau M. Thrombotic microangiopathy and purtscher-like retinopathy as a rare presentation of juvenile dermatomyositis. Pediatrics. (2012) 129:821–4. 10.1542/peds.2011-0338 22311994

[12] Nuñez Zuno JA Khaddour K. Thrombotic thrombocytopenic purpura evaluation and management. In: StatPearls. Treasure Island, FL: StatPearls Publishing (2020). Available online at: https://www.ncbi.nlm.nih.gov/books/NBK470585/?report=reader#_NBK470585_pubdet_ 29261870

[13] Moore J Hayward C Warkentin T Kelton J. Decreased von Willebrand factor protease activity associated with thrombocytopenic disorders. Blood. (2001) 98:1842–6. 10.1182/blood.V98.6.1842 11535519

[14] Tsai HM. Pathophysiology of thrombotic thrombocytopenic purpura. Int J Hematol. (2010) 91:1–19. 10.1007/s12185-009-0476-1 20058209 PMC3159000

[15] Masias C Cataland SR. Novel therapies in thrombotic thrombocytopenic purpura. Res Pract Thromb Haemost. (2017) 2:19–26. 10.1002/rth2.12066 30046703 PMC6055500

[16] Gürcan HM Keskin DB Stern JN Nitzberg MA Shekhani H Ahmed AR. A review of the current use of rituximab in autoimmune diseases. Int Immunopharmacol. (2009) 9:10–25. 10.1016/j.intimp.2008.10.004 19000786

[17] Wiestner A Cho HJ Asch AS Michelis MA Zeller JA Peerschke EIB. Rituximab in the treatment of acquired factor VIII inhibitors. Blood. (2002) 100:3426–8. 10.1182/blood-2002-03-0765 12384448

[18] Emer JJ Claire W. Rituximab: a review of dermatological applications. J Clin Aesthet Dermatol. (2009) 2:29–37. 20729962 PMC2924133

[19] Agrawal A McKibbin MA. Purtscher's and purtscher-like retinopathies: a review. Surv Ophthalmol. (2006) 51:129–36. 10.1016/j.survophthal.2005.12.003 16500213

[20] Yan Y Xi S. Purtscher like retinopathy associated with dermatomyositis. BMC Ophthalmol. (2013) 13:36. 10.1186/1471-2415-13-36 23883070 PMC3724690

[21] Papadopoulou C McCann LJ. The vasculopathy of juvenile dermatomyositis. Front Pediatr. (2018) 6:284. 10.3389/fped.2018.00284 30356795 PMC6189418

